# Anthracene induces oxidative stress and activation of antioxidant and detoxification enzymes in *Ulva lactuca* (Chlorophyta)

**DOI:** 10.1038/s41598-021-87147-5

**Published:** 2021-04-08

**Authors:** Alberto González, Constanza Vidal, Daniela Espinoza, Alejandra Moenne

**Affiliations:** Laboratory of Marine Biotechnology, Faculty of Chemistry and Biology, University of Santiago of Chile, Alameda, 3363 Santiago, Chile

**Keywords:** Environmental biotechnology, Plant biotechnology, Plant physiology, Plant stress responses

## Abstract

In order to analyze whether the marine macroalga *Ulva lactuca* can absorb and metabolize anthracene (ANT), the alga was cultivated with 5 µM ANT for 0–72 h, and the level of ANT was detected in the culture medium, and in the alga. The level of ANT rapidly decreased in the culture medium reaching a minimal level at 6 h, and rapidly increased in the alga reaching a maximal level at 12 h and then decreased to reach a minimal level at 48 h of culture. In addition, ANT induced an increase in hydrogen peroxide that remained until 72 h and a higher increase in superoxide anions that reach a maximal level at 24 h and remained unchanged until 72 h, indicating that ANT induced an oxidative stress condition. ANT induced an increase in lipoperoxides that reached a maximal level at 24 h and decreased at 48 h indicating that oxidative stress caused membrane damage. The activity of antioxidant enzymes SOD, CAT, AP, GR and GP increased in the alga treated with ANT whereas DHAR remained unchanged. The level of transcripts encoding these antioxidant enzymes increased and those encoding DHAR did not change. Inhibitors of monooxygenases, dioxygenases, polyphenol oxidases, glutathione-*S*-transferases and sulfotransferases induced an increase in the level of ANT in the alga cultivated for 24 h. These results strongly suggest that ANT is rapidly absorbed and metabolized in *U. lactuca* and the latter involves Phase I and II metabolizing enzymes.

## Introduction

Polycyclic aromatic hydrocarbons (PAHs) are molecules commonly formed by two to six benzene rings that can be arranged in linear, angular or clustered forms. Anthracene (ANT) is formed by three benzene rings arranged linearly, phenanthrene (PHE) is constituted by three benzene rings arranged in angle, and both are considered a small PAH^1^. Benzo[a]pyrene (BaP) is constituted by five benzene rings arranged as a cluster and correspond to a large PAH. PAHs are found in water and soil, they are present in crude oil and industrial effluents and they are produced by fossil fuel combustion, oil refining and automobile emissions^1^. PAHs are hydrophobic compounds that are very stable and persistent in the environment and, thus, magnified in the food chain^1^. PAHs are sensitive to sunlight, mainly to UV light, and their derivatives are quinones and hidroxyquinones^2,3^. PAHs are toxic to animals and plants since they interfere with function of membranes and with proteins and enzymes located in cellular membranes^1^. In Chile, there are several so-called sacrifice zones located in coastal sites displaying an accumulation of oil refineries and thermo-electric industries, in northern, central and southern Chile and displayed heavy metals and polycyclic aromatic hydrocarbons pollution^4,5^. In these costal zones successive crude oil spillages have occurred in seawater producing a detrimental effect in human health and in biodiversity^4,5^. Thus, it is urgently required to find marine species that are capable to bioremediate seawater from crude oil and PAHs.

Regarding PAHs and plants, the aquatic monocot plant *Lemna gibba* (duckweed) cultivated with 2 µg mL^−1^ of ANT, PHE and BaP for 24 h showed an inhibition of growth and chlorophyll content, and the absorption of ANT, PHE and BaP of 58%, 66% and 33%, respectively, after 24 h of culture^6^. In addition, ANT, PHE and BaP exposed to sunlight and UV light showed that its photoxidized derivatives induced a stronger inhibition of growth than the native compounds, and the toxicity of ANT is higher than PHE and higher than BAP in *L. gibba*^2,3^. ANT, PHE and BAP exposed to sunlight showed derivatives that are mainly quinones and hydroxyquinones, but also smaller derivatives such as phthalic acid, salicylic acid and benzoic acid^7,8^. In plants and animals, PAHs can also be degraded at intracellular level by monooxygenases and dioxygenases (Phase I) producing hydroxyl derivatives and hydroxydiols, respectively, and that can be further conjugated with glutathione (GSH), glucose, maltose or glucuronic acid by metabolizing enzymes such as glutathione-*S*-transferases (GSTs), glucosyl transferases and glucuronyl-transferases, or derivatized with a sulfate, methyl or acetyl group (Phase II) by sulfotransferases, methyltransferases and acetyltransferases. Phase I and Phase II are required to increase solubility of PAHs in water allowing their further degradation and/or excretion in plants and animals^9,10^.

Regarding PAHs and other hydrocarbons degradation in plants, the tall fescue plant *Festuca arundinacea* cultivated with 1 µg mL^−1^ of ANT for 16 days showed the degradation of ANT to anthrone, anthraquinone and hydroanthraquinone that were mainly accumulated in root cells in organelles and cell soluble fraction^11^. These results are similar to those previously obtained in roots of wheat and tomato exposed to ANT^12^. The aquatic liverwort *Riccia fluitans* cultivated with 0.5–10 µM of PHE for 96 h showed an oxidative stress condition, a decrease in chlorophyll and carotenoid contents, a decrease in photosynthesis efficiency and an increase in transcripts encoding antioxidant enzymes and enzymes involved in polyamine synthesis^13^. In this sense, it is important to point out that polyamines putrescine, spermine and spermidine are involved in protection of cellular and organellar membranes. *A. thaliana* plants cultivated with 0–250 µM of PHE showed an oxidative stress condition and the increase in the level of transcripts encoding antioxidant enzymes superoxide dismutase (SOD), catalase (CAT) and ascorbate peroxidase (AP) and in the level of transcripts encoding 14 different GSTs, glucosyl- and glucuronyl-transferases, and enzymes involved in polyamines synthesis^14^. Maize plants cultivated soil contaminated with 1, 2.5 and 5% of petroleum for 30 days showed an increase in the level of transcripts of five GSTs, several-cysteine proteases, and enzymes involved in N assimilation and antioxidant enzymes such as SOD, AP, CAT and glutathione reductase (GR)^15^. Thus, GSTs and glycosyltransferases may be involved in the solubilization of PAHs and other hydrocarbons in plants.

Regarding PAHs degradation and toxicity in microalgae, the green microalga *Selenestartum capricornutum* cultivated with ANT showed an inhibition of growth, a IC_50_ of 16 µg L^−1^, and the toxicity of ANT increased with the exposure of the alga to UV-A light^16^. In addition, *S. capricornutum* exposed to BAP displayed the formation of dihydrodiols, mainly 9,10-dihydrodiol, and the exposure to UV-A light increased the level of dihydrodiols and decreased the level of quinones^17^. The green microalga *S. armatus* cultivated with ANT and PHE displayed an oxidative stress condition and the increase in activity of the antioxidant enzyme SOD^18^. The green microalga *C. reinhardtii* cultivated with 0.7–4.2 µM of ANT for 24 h displayed an inhibition of growth, a decrease in chlorophyll fluorescence and in the efficiency of photosystem II (PSII) showing a EC50 of 1.6 µM^19^. The green microalga *Tetraselmis chuii* exposed to ANT, PHE and naphthalene (NAPH) for 96 h showed an inhibition of growth, the toxicity was PHE > ANT > NAPH and toxicity of these PAHs was enhanced by the increase in 5 °C of temperature^20^. Recently, it was shown that the microalga *Euglena agilis* cultivated with increasing concentrations of ANT, ranging from 0.35 to 84 µM, for 96 h showed a decreased in growth, in photosynthetic efficiency and in the content of chlorophylls and carotenoids^21^.

Regarding PAHs and other hydrocarbons degradation in marine algae, the green macroalgae *Ulva intestinalis* and *Cladophora glomerata* metabolize 42–49% of BaP in 6 h^22^. The green macroalga *Ulva lactuca* exposed to gasoline containing paraffinic, isoparaffinic, aromatic, naphthenic and olefinic hydrocarbons, constituted by 5–10 C, showed a decrease in chlorophylls, carotenoids and polyphenols levels and an increase in soluble sugars and starch^23^. Recently, it was shown that *U. lactuca* exposed to 5 µM of BaP rapidly absorbed and metabolized BaP since 79% of the compound was degraded after 72 h^24^. The alga displayed an oxidative stress condition reflected in the increase in reactive oxygen species (ROS), mainly superoxide anions, and an increase in activities of antioxidant enzymes such as ascorbate peroxidase (AP), catalase (CAT), glutathione reductase (GR) and glutathione peroxidase (GP) and the metabolizing enzyme GST^24^. The level of transcripts encoding these antioxidant and metabolizing enzymes were increased in response to BaP suggesting that the increase in activities is due to the enhanced expression of genes encoding these enzymes^24^. Furthermore, the alga treated with inhibitors of CYP450 and in the synthesis of GSH showed an increase in intracellular BaP level suggesting that CYP450 and GST are involved in Phase I and II of BaP detoxification^24^. Thus, the marine alga *U. lactuca* can use other hydrocarbons and PAHs as alternative sources of C.

In this work, we analyze the ability of *U. lactuca* to absorb and degrade ANT, the induction of an oxidative stress condition, the activation of antioxidant enzymes and the level of transcripts encoding these enzymes. Furthermore, we analyze the effects of inhibitors of Phase I detoxification enzymes such as monooxygenases and dioxygenases and Phase II detoxification enzymes such as polyphenol oxidases (PPOs), GSTs and sulfotransferases (STs), and the level of ANT was determined in *U. lactuca*.

## Results

### Viability of *U. lactuca* exposed to increasing concentrations of ANT

**Figure 1:**
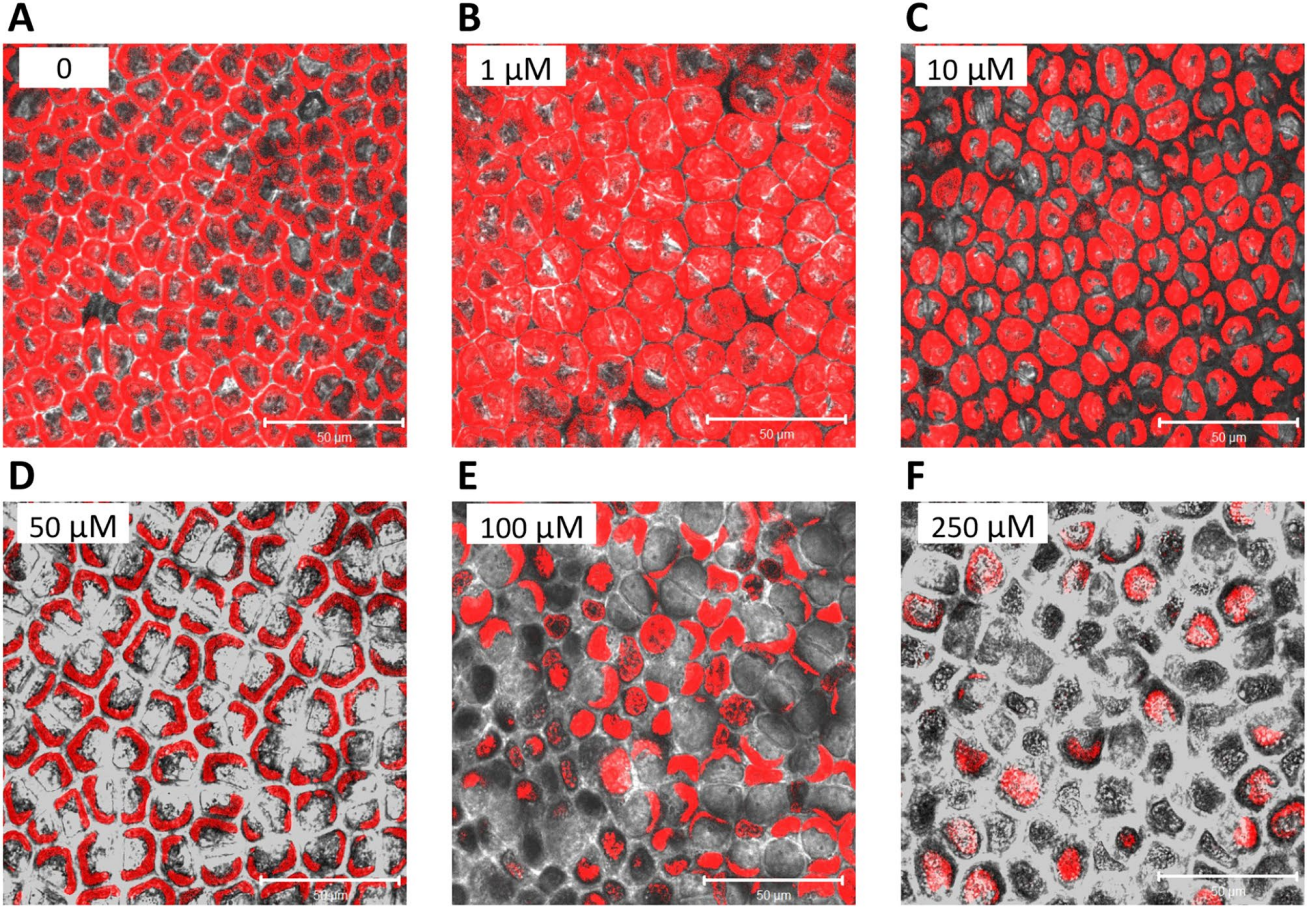
Visualization of *U. lactuca* cells cultivated with 0–250 μM of anthracene for 7 days. The viability of the cells was analyzed based on the red autofluorescence of chlorophylls in chloroplasts by confocal microscopy.

In order to determine a sub-lethal concentration of ANT in *U. lactuca*, the alga was cultivated in artificial seawater without ANT (control) and with increasing concentrations of ANT corresponding to 1, 10, 50, 100 and 250 µM, for 7 days (Fig. 1). Cell viability was analyzed through visualization of chlorophyll fluorescence in chloroplasts using confocal microscopy. Chlorophyll fluorescence in the algae treated with 10 μM of ANT was similar to the control indicating full viability (Fig. 1A,B). Algae treated with 50–250 µM of ANT showed a progressive decrease in chlorophyll fluorescence compared to the control (Fig. 1C–F). Thus, the chosen sub-lethal concentration of ANT for further experiments was 5 µM.

### ANT is rapidly incorporated and metabolized in *U. lactuca*

**Figure 2:**
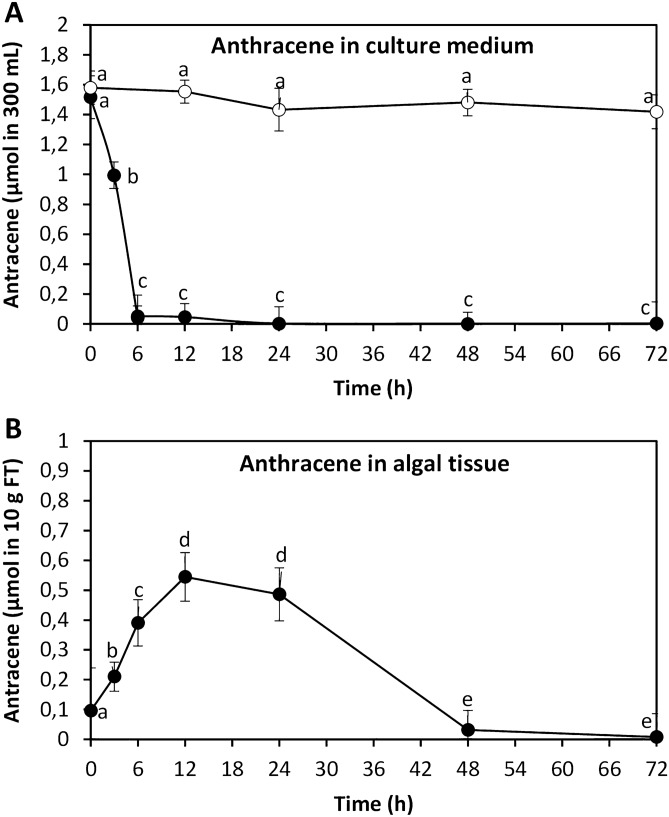
Level of anthracene (ANT) in the culture medium (**A**) and in *U. lactuca* (**B**) cultivated with 1.6 μmol of ANT in 300 mL of culture medium (5 μM) for 0–72. Black circles represent the level of ANT in the culture with ANT and open circles represent the level of ANT in the culture medium with ANT but without the alga. Symbols represent mean values of three independent experiments $\pm$ SD. Letters indicate significant differences among experiments (*P* < 0.05).

To determine whether *U. lactuca* can incorporate and metabolize ANT, the alga was cultivated in without ANT (control) and with 5 μM of ANT for 0–72 h, and the level of ANT was determined in the culture medium and in the alga. The level of ANT in seawater was 5 µM, which corresponds to 1.6 µmol in 300 mL of seawater, and it rapidly decreased reaching a minimal level at 6 h of culture corresponding to 0.05 µmol in 300 mL of seawater or 1,6 µM of ANT and almost completely disappeared at 48 h of culture (Fig. 2A) while the amount of ANT in the culture medium without the alga remained constant until the end of the experiment. The level of ANT in the alga rapidly increased reaching a maximal level of 0.54 µmol in 10 g of algal tissue at 12 h of culture and then decreased reaching a minimal level of 0.03 µmol in 10 g of algal tissue at 48 h of culture (Fig. 2B). These results indicate that half of the 5 µM ANT was absorbed by the alga at 6 h culture and almost totally absorbed at 48 h. Thus, ANT is rapidly incorporated in *U. lactuca.*

### ANT induced an oxidative stress condition in *U. lactuca*

**Figure 3:**
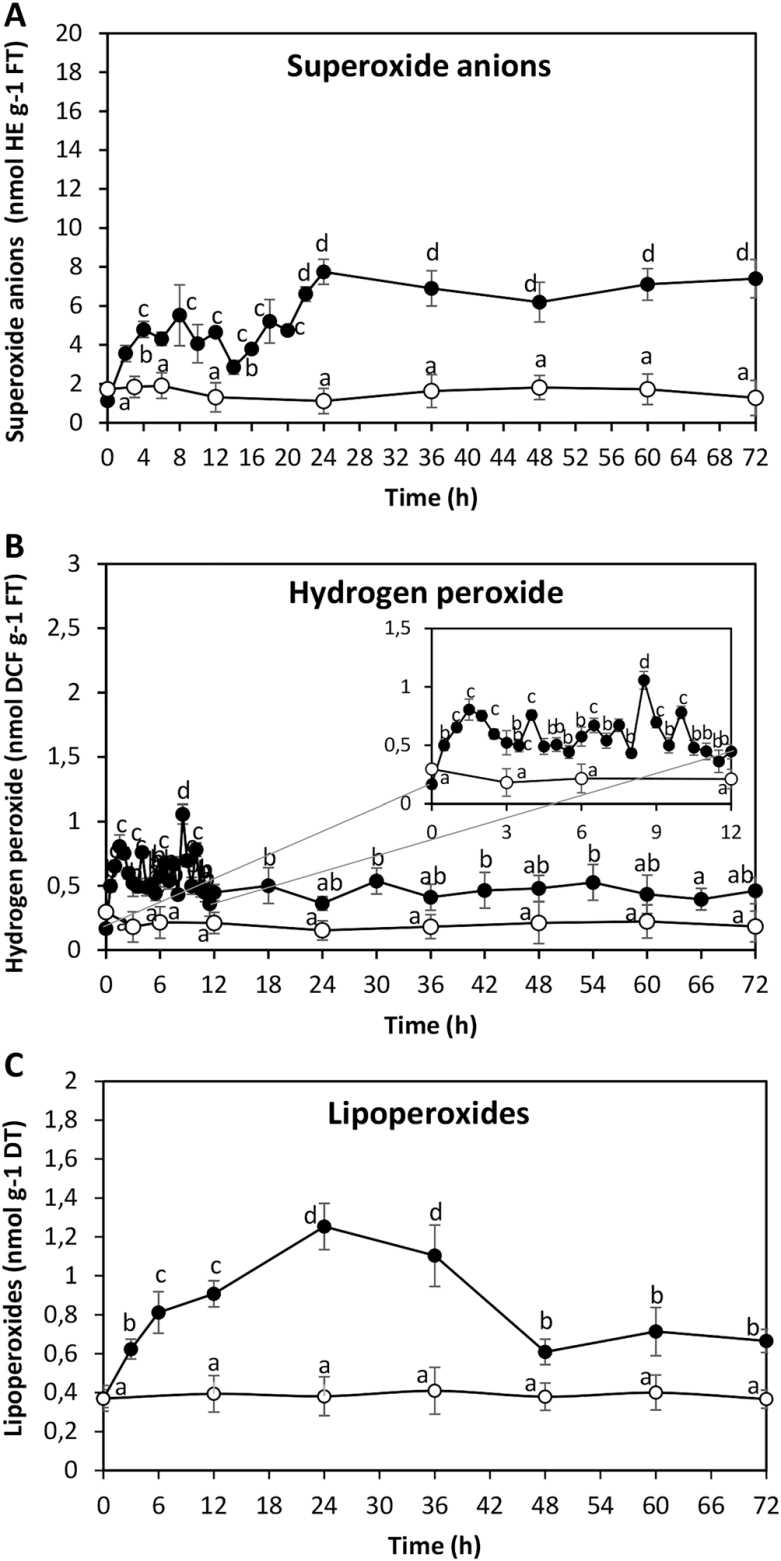
Level of superoxide anions (**A**), hydrogen peroxide (**B**) and lipoperoxides (**C**) in *U. lactuca* cultivated without ANT (open circles) and with 5 μM of ANT (black circles) for 0–72 h. The level of superoxide anions and hydrogen peroxide are expressed as nanomole per gram of fresh tissue (FT) and lipoperoxides in nanomole per gram of dry tissue (DT) and time in hours. The subframe represents the level of hydrogen peroxide from 0 to 12 h of culture. Symbols represent mean values of three independent experiments $\pm$ SD. Letters indicate significant differences among experiments (*P* < 0.05).

To analyze whether ANT induce oxidative stress in *U. lactuca*, the alga was cultivated without ANT (control) and with 5 µM of ANT for 0–72 h, and the level of superoxide anions, hydrogen peroxide and lipoperoxides was determined. The level of superoxide anions increased reaching a maximal level of 7.75 nmol g^−1^ of fresh tissue (FT) at 24 h of culture and remained increased until 72 h (Fig. 3A). The level of hydrogen peroxide increased reaching a maximal level of 0.8 nmol g^−1^ of FT at 1.5 h, 1.1 nmol g^−1^ of FT at 8.5 h and 0.78 at 10 h of culture and remained increased until 72 h (Fig. 3B), The level of lipoperoxides increased reaching a maximal level of 1.25 µmol g^−1^ of dry tissue (DT) at 24 h of culture, decreased to 0.61 µmol at 48 h, and remained stable until 72 h (Fig. 3C). Thus, ANT induced an oxidative stress condition and membrane damage in *U. lactuca*.

### ANT increased antioxidant enzymes activities in *U. lactuca*

**Figure 4:**
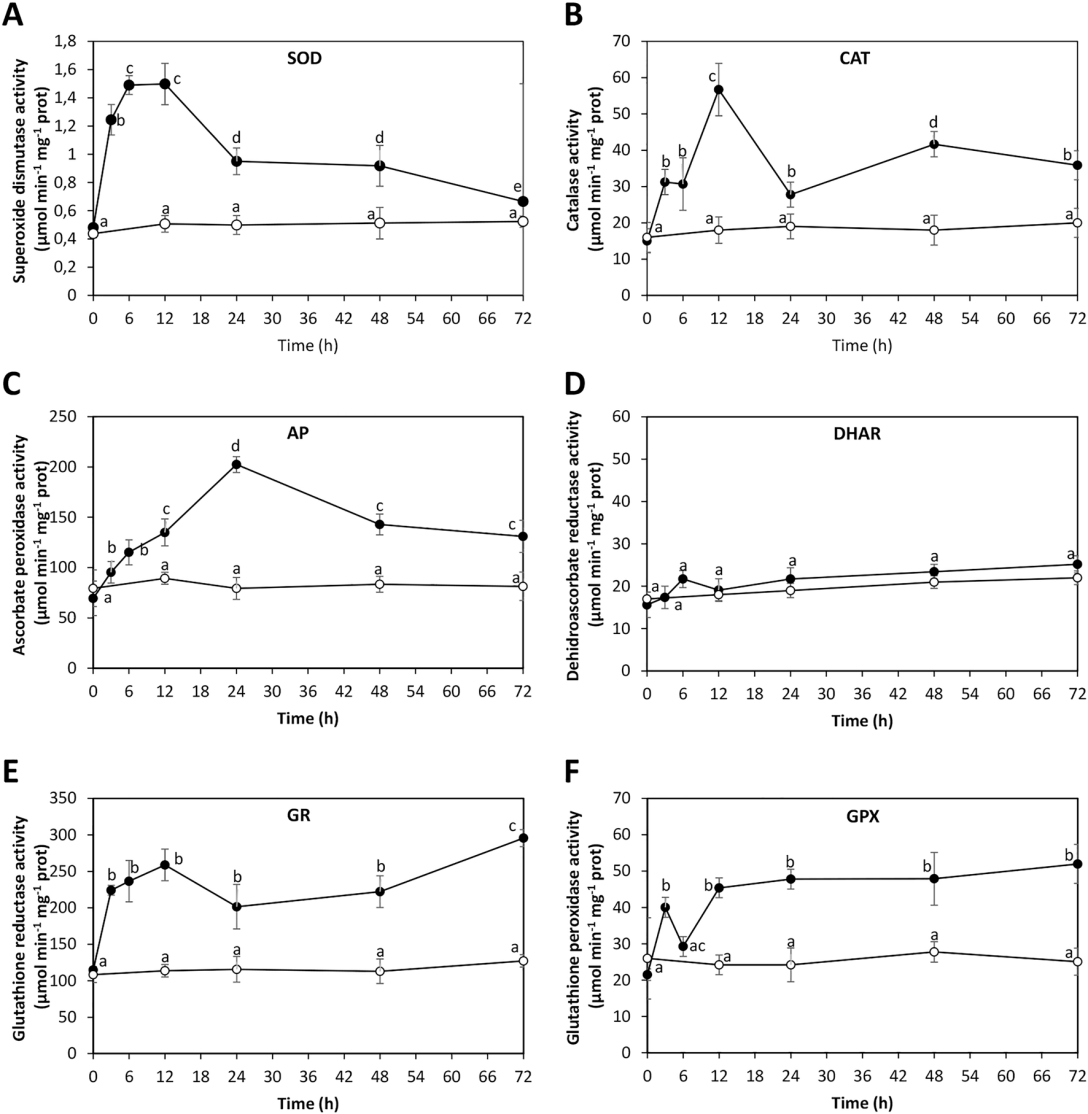
Activities of antioxidant enzymes superoxide dismutase (**A**), catalase (**B**), ascorbate peroxidase (**C**), dehydroascorbate reductase (**D**), glutathione reductase (**E**) and glutathione reductase (**F**) in *U. lactuca* cultivated without anthracene (open circles) and with 5 μM anthracene (black circles) for 0–72 h. Activities of antioxidant enzymes are expressed as micromole per minute per gram of fresh tissue (FT) and time in hours. Symbols represent mean values of three independent experiments $\pm$ SD. Letters indicate significant differences among experiments (*P* < 0.05).

To analyze whether the oxidative stress condition induced the activation of antioxidant enzymes, the alga was cultivated without ANT (control) and with 5 µM of ANT for 0–72 h, and the activities of antioxidant enzymes superoxide dismutase (SOD), catalase (CAT), ascorbate peroxidase (AP), dehydroascorbate reductase (DHAR), glutathione reductase (GR) and glutathione peroxidase (GP) were determined. The activity of SOD was 0.48 µmol of superoxide anions consumed per min^−1^ mg^−1^ of protein in control condition and it remained unchanged until 72 h of culture (Fig. 4A). SOD activity increased in treated alga from 0.48 to 1.5 µmol min^−1^ mg^−1^ of protein at 6 and12 h of culture and decreased to 0.7 µmol min^−1^ mg^−1^ of protein at 72 h (Fig. 4A). The activity of CAT was 15 µmol of hydrogen peroxide consumed per min^−1^ mg^−1^ of protein in control condition and it remained unchanged until 72 h (Fig. 4B). CAT activity increased in treated alga from 15 to 58 µmol min^−1^ mg^−1^ of protein 12 h of culture and decreased to 28 µmol min^−1^ mg^−1^ of protein at 24 h and increased again to 36 µmol min^−1^ mg^−1^ of protein at 72 h (Fig. 4B). The activity of AP was 69 µmol min^−1^ mg^−1^ of protein in control condition and it remained unchanged until 72 h (Fig. 4C). AP activity increased in treated alga from 69 to 202 µmol of ascorbate consumed per min^−1^ mg^−1^ of protein at 24 h of culture and decreased to 131 µmol min^−1^ mg^−1^ at 72 h (Fig. 4C). The activity of DHAR was 16 µmol of dehydroascorbate consumed per min^−1^ mg^−1^ of protein and it did not change in control or treated conditions (Fig. 4D). The activity of GR was 115 µmol of glutathione consumed per min^−1^ mg^−1^ of protein in control condition and it remained unchanged until 72 h of culture (Fig. 4E). GR activity increased from 115 to 259 µmol min^−1^ mg^−1^ of protein at 12 h, decreased to 202 µmol min^−1^ mg^−1^ of protein at 24 h and increased again to 296 µmol min^−1^ mg^−1^ of protein at 72 h (Fig. 4E). The activity of GP was 22 µmol of glutathione consumed per min^−1^ mg^−1^ of protein in control condition and it did not change until 72 h of culture (Fig. 4F). GP activity increased from 22 to 40 µmol min^−1^ mg^−1^ of protein at 3 h of culture, decreased to 29 µmol min^−1^ mg^−1^ of protein at 6 h of culture, increased to 45 µmol min^−1^ mg^−1^ of protein at 12 h and remained unchanged until 72 h of culture (Fig. 4F). Thus, the oxidative stress condition induced by ANT is buffered by the activation of several antioxidant enzymes in *U. lactuca*. The maximal activity of antioxidant enzymes occurred at 12 h of culture, except for AP that reached a maximal level at 24 h of culture.

### ANT increased the level of transcripts of antioxidant enzymes in *U. lactuca*

**Figure 5:**
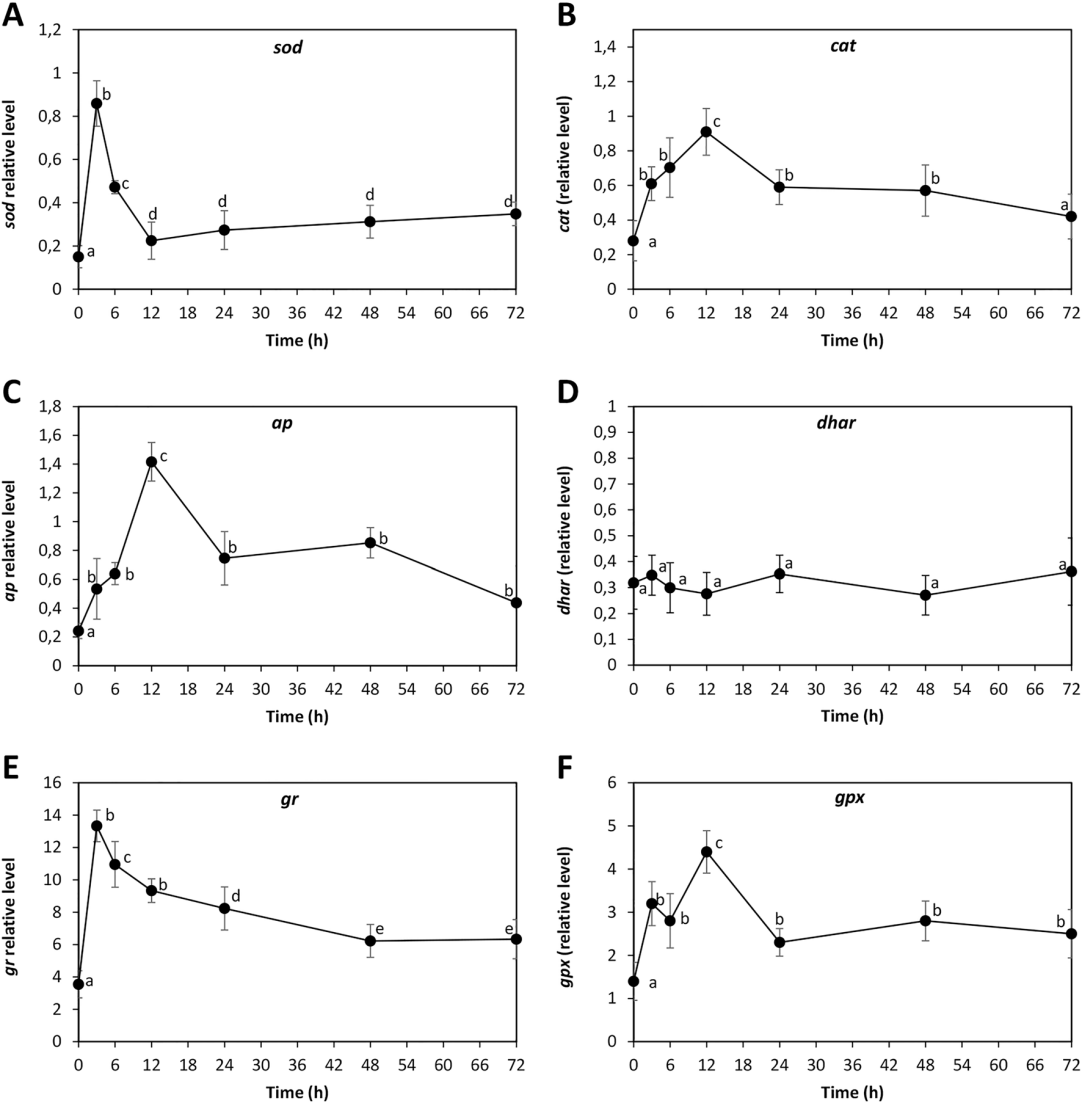
Relative level of transcripts encoding antioxidant enzymes superoxide dismutase (**A**), catalase (B), ascorbate peroxidase (**C**), dehydroascorbate reductase (**D**), glutathione reductase (**E**) and glutathione reductase (**F**) in *U. lactuca* cultivated with 5 μM anthracene for 0–72 h (black circles). The relative level of transcripts is expressed as 2^−ΔΔCT^. Symbols represent mean values of three independent experiments $\pm$ SD. Letters indicate significant differences among experiments (*P* < 0.05).

In order to analyze whether the increase in activities of antioxidant enzymes is due to an increase in their expression, the alga was cultivated without ANT (control) and with 5 μM of ANT for 0–72 h, and the relative levels of transcripts encoding antioxidant enzymes SOD, CAT, AP, DHAR, GR and GP were determined. The relative level of transcripts of SOD increased to reach a maximal level of 0.86 times of increase at 3 h of culture, decreased to 0.22 times at 12 h and slightly increased to 0.35 times at 72 h of culture (Fig. 5A). The relative level of transcripts encoding CAT increased to reach a maximal level of 0.91 times of increase at 12 h of culture and decreased to reach 0.42 times of increase at 72 h of culture (Fig. 5B). The relative level of transcripts encoding AP increased to reach a maximal level of 1.42 times of increase at 12 h of culture and decreased to 0.75 times at 24 h and to 0.44 times at 72 h of culture (Fig. 5C). In contrasts, the relative level of transcripts encoding DHAR did not change from 0 to 72 h of culture (Fig. 5D). The relative level of transcripts encoding GR increased to reach a maximal level of 13.3 times of increase at 12 h of culture and decreased to reach 6.3 times of increase at 72 h (Fig. 5E). The relative level of transcripts encoding GP increase to reach a maximal level of 4.4 times of increase at 12 h of culture and decreased to reach 2.5 times at 72 h (Fig. 5F). Thus, the increase in the level of transcripts encoding antioxidant enzymes occurred mainly at 12 h of culture, except those encoding SOD that reached a maximal level at 3 h of culture indicating that the increase in antioxidant enzyme activities is due, at least in part, to the increase in their expression.

### ANT induced an increase in activity and expression of GST metabolizing enzyme

**Figure 6:**
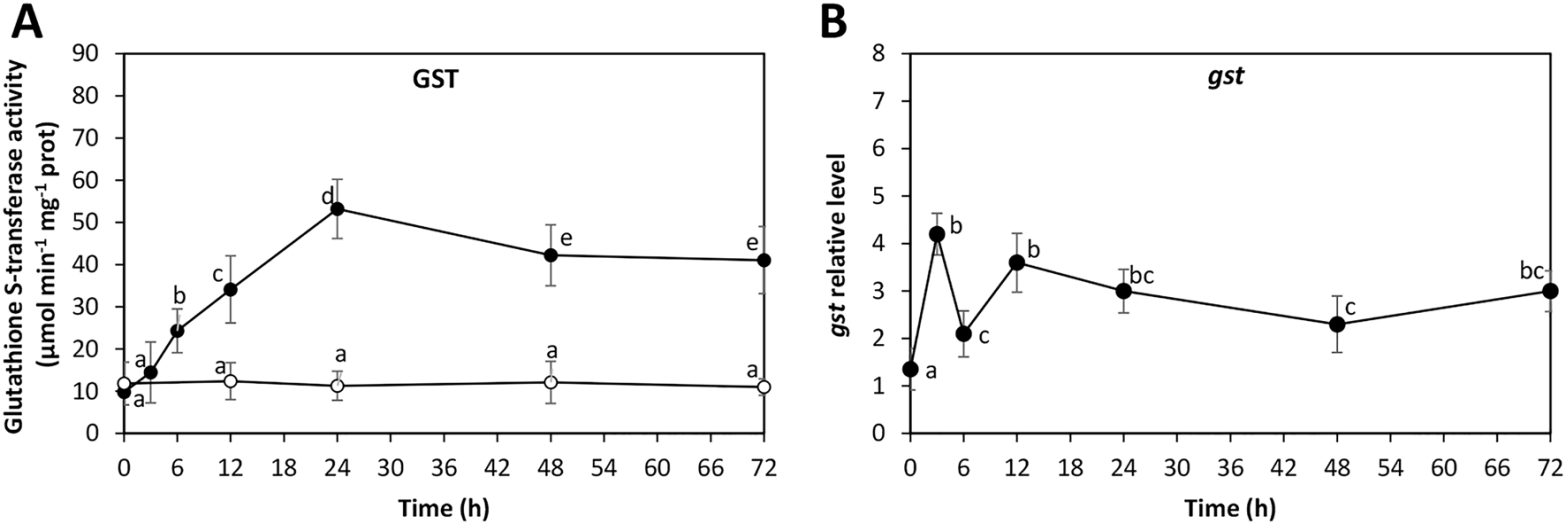
Activity of glutathione-*S*-transferase (GST, A) in *U. lactuca* cultivated without anthracene (open circles) and with 5 μM of ANT (black circles) for 0–72 h. The activity of GST is expressed as micromole per gram of fresh tissue (FT). Relative level of GST transcripts (**B**) in the alga cultivated with 5 μM of ANT (black circles). The relative level of GST transcripts is expressed as 2^−ΔΔCT^. Symbols represent mean values of three independent experiments $\pm$ SD. Letters indicate significant differences among experiments (*P* < 0.05).

In order to analyze the mechanisms involved in ANT detoxification, the alga was cultivated without ANT (control) and with 5 µM of ANT for 0–72 h, and the activity of the metabolizing enzyme GST and the relative level of transcripts encoding GST were determined. The activity of GST in control condition was 9.8 µmol min^−1^ mg^−1^ of protein and it remained unchanged until 72 h of culture (Fig. 6A). GST activity in treated algae increased from 9.8 to 53 µmol min^−1^ mg^−1^ of protein at 24 h and decreased to 41 µmol min^−1^ mg^−1^ of protein at 72 h of culture (Fig. 6A). The level of transcripts encoding GST increased in 4.2 times at 3 h of culture, decreased to 2.1 times at 6 h, increased to 3.6 times at 12 h and slightly decreased to 3 times at 72 h of culture (Fig. 6B). Thus, ANT induce an increase in the activity of the detoxification enzyme GST and this increase in activity is due, at least in part, to the increase in its expression.

### ANT induced an increase in Phase I and II metabolizing enzymes activities

**Figure 7:**
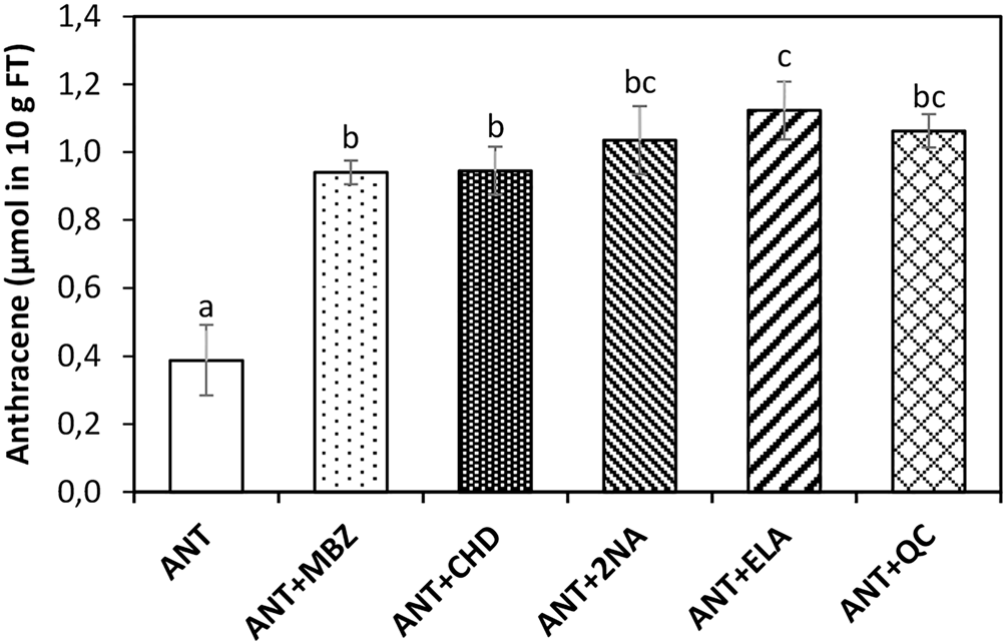
Level of anthracene in *U. lactuca* cultivated with 5 μM of ANT for 24 h (ANT, open bar) and with 0.5 μM of mebendazole (MBZ), an inhibitor of monooxygenases, cyclohexanedione (CHD), an inhibitor of dioxygenases, 2-naphtoic acid (2-NA), an inhibitor of polyphenol oxidases, ellagic acid (ELA), an inhibitor of GST, and quercetin (QC), an inhibitor of sulfotransferases, and with 5 µM of ANT for 24 h (dashed bars). The level of ANT is expressed as micromole in 10 g of algae fresh tissue (FT). Bars represent mean values of three independent experiments. Letters indicate significant differences among experiments (*P* < 0.05).

In order to analyze the involvement of Phase I and II detoxification enzymes, the alga was cultivated with 5 μM of ANT (control) and 0.5 µM inhibitors of Phase I enzymes CYP450-dependent monooxygenases and dioxygenases, and phase II enzymes PPOs, GSTs and STs and with 5 µM ANT for 24 h, and the amount of ANT in the alga was determined (Fig. 7). The level of ANT in control algae was 0.4 µmol in 10 g of FT and in the alga treated with inhibitors of monooxygenases, dioxygenases, PPO, GST and sulfotransferases it was 0.9, 0.9, 1, 1.1 and 1.1 µmol in 10 g of FT, respectively, which represents increases of 36, 36, 43, 48 and 44% compare to the control (Fig. 7). The level of ANT in the alga increased with all inhibitors, mainly with those that inhibit PPOs, GSTs and STs (Fig. 7). Thus, Phase I enzymes, monooxygenases and dioxygenases, and Phase II enzymes, PPOs, GSTs and STs are involved in ANT detoxification in *U. lactuca.*

## Discussion

In this work, we showed that ANT is rapidly incorporated and metabolized in the marine alga *U. lactuca* reaching 46% of metabolization at 6 h of culture and 98% at 48 h. These results are accord with those previously obtained in the green macroalgae *U. intestinalis* and *C. glomerata* that metabolized 42–49% of BaP in 6 h^22^. This contrasts with results obtained in brown macroalgae such as *Fucus vesiculosus* and *Chorda filum* that metabolized only 5% of BaP after 4 days^22^. Thus, green macroalgae appeared to be more efficient than brown macroalgae to metabolize PAHs. Recently, it has been shown that *U. lactuca* metabolized 68% of BaP at 24 h and 75% of BaP at 72 h of culture^24^. Thus, *U. lactuca* metabolized more efficiently the three-ring ANT than the five-ring BaP but, interestingly, the alga can metabolize both PAHs.

ANT induced an oxidative stress condition in *U. lactuca* characterized by the accumulation of ROS, mainly superoxide anions. In addition, ANT increased the level of lipoperoxides with a maximal level of 1.25 µmol g^−1^ of DT at 24 h of culture which indicate that ROS induced membrane damage in the alga. These results are in accord with those obtained in *U. lactuca* cultivated with 5 µM of BaP that showed an increase in ROS, mainly superoxide anions, and an increase in lipoperoxides showing a maximal level of 0.68 µmol g^−1^ of DT at 6 and 12 h of culture^24^. Thus, it appeared that ANT induced a higher increase of oxidative stress than BaP reflected by membrane damage in *U. lactuca*. In this sense, it has been shown that ANT induced a higher inhibition of growth and a higher chlorosis than BaP in *L. gibba*^2,3^. Thus, it is possible that the higher toxicity of ANT compared to BaP is due to the induction of a higher membrane damage which may affect mainly chloroplasts inducing chlorosis. The aquatic liverwort *R. communis* cultivated with 0–10 μM of PHE for 72 h showed an increase in amino acid leakage from 2.5 to 10 µM and an increase in liperoxides and carbonylated proteins with 0.5 µM of PHE indicating that PHE also causes an oxidative stress condition and membrane damage^13^. Thus, PAHs cause oxidative stress and membrane damage in plants and algae and ANT is more toxic than BaP.

ANT induced an increase in activities of antioxidant enzymes SOD, CAT, AP, GR and GP, but not in DHAR. The activation of enzyme antioxidant system may participate in the buffering of ANT-induced oxidative stress. These results are in accord with those obtained in the aquatic plant *R. fluitans* cultivated with 0.5 μM of PHE for 96 h that showed an oxidative stress condition and the increase in activities of the antioxidant enzymes AP and GR^13^. *A. thaliana* plants cultivated with PHE also showed an oxidative stress condition and the increase in the level of transcripts encoding SOD, AP and GR^14^. Maize plants cultivated soil contaminated with 1–5% of petroleum for 30 days showed an increase in the level of transcripts of antioxidant enzymes such as SOD, AP, CAT and GR^15^. It has been recently shown that *U. lactuca* cultivated with BaP for 72 h showed an oxidative stress condition and the activation of antioxidant enzymes SOD, CAT, AP, GR and GP, but not DHAR^24^. Thus, plants and marine alga exposed to PAHs displayed an oxidative stress condition that is partially buffered by the activation by antioxidant enzymes. As in the case of *U. lactuca* and BaP, the alga cultivated with ANT did not show and increase in DHAR activity indicating that the Halliwell-Asada-Foyer cycle is uncoupled^24,25^. DHAR activity uses GSH as substrate and produces oxidized GSH (GSSG) as product^24^. On the other hand, it was observed that GR and GP showed the higher increase in activities in *U. lactuca* cultivated with ANT and BaP and, it is important to note, that both enzymes consume GSH. Thus, it is possible that the lack of DHAR activity is due to the high consumption of GSH by enzymes such as GR and GP. Thus, an important molecule for PAHs tolerance in *U. lactuca* is the potential synthesis and the consumption of GSH.

Interestingly, the level of transcripts of antioxidant enzymes SOD, CAT, AP, GR and GP increased in *U. lactuca* exposed to ANT, but not those encoding DHAR. The increase in the level of transcript of the latter antioxidant enzymes showed maximal levels that are previous to maximal enzyme activities indicating that the increase in activities is due, at least in part, to their enhanced expression. In addition, the level of transcripts of the metabolizing enzyme GST increased with a maximal level at 3 and 12 h and GST activity showed a maximal level at 24 h. Thus, the increase in GST metabolizing activity is also transcriptionally regulated. It is important to mention that GST also consumes GSH, as GR and GP, and this is another metabolic point of GSH consumption which may also explain the absence of DHAR activity (see above). It was previously shown that BaP also increase activity and expression of GST in *U. lactuca*^24^. The involvement of GST in the detoxification of PAHs and other hydrocarbons also occurred in plants since *A. thaliana* treated with PHE that showed an increase in the level of transcripts encoding 14 different GSTs^14^ and maize plants cultivated soil contaminated with petroleum showed an increase in the level of transcripts of five GSTs^15^. Thus, GSTs are key enzymes in degradation of PAHs and other hydrocarbons in plants and algae.

Interestingly, inhibitors of Phase I enzymes, CYP450-dependent monooxygenases and dioxygenases, and Phase II enzymes, PPOs, GSTs and STs are involved in ANT detoxification in *U. lactuca* mainly those that inhibit PPOs, GSTs and STs. In this sense, it has been shown in the green macroalgae *E. intestinalis* and *C. glomerata* metabolize BaP through activation of phenol oxidase activity and peroxidase^22^. In addition, GSTs are involved in PHE detoxification in willow trees (*Salix viminalis*) showing and increase in the level of GST transcripts and in those coding for enzymes involved in GSH synthesis^26^. GST activity was also increased in common beans (*Phaseolus vulgaris*) exposed to ANT and fluorene^27^. Regarding STs in plants, *A. thaliana* genome encode 21 ST genes and rice genome encode 29 functional genes and STs are involved in sulfation of hydroxyl groups present in flavonoids, phenolic acids, terpenes, gibberellic acids and glucosinolates, and in the detoxification of hydroxylated xenobiotics^28^. In genomes of red and brown micro- and macroalgae, several potential genes of STs have been detected but their substrate preference has not yet been identified^29^. Thus, plants green macroalgae exhibited Phase I and II enzymes activities that may be involved in PAHs detoxification.

## Conclusion

**Figure 8:**
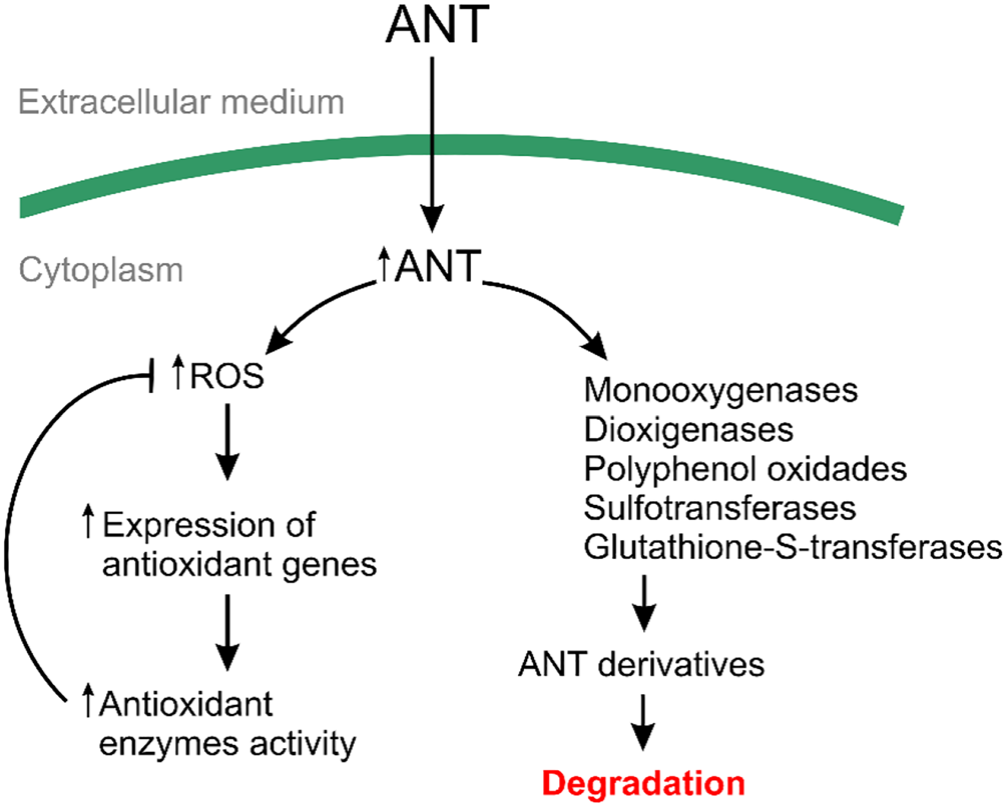
Model of anthracene detoxification in the marine alga *U. lactuca*. The alga cultivated with ANT rapidly incorporate and degrade this molecule showing an increase in ANT in the alga. ANT induces an oxidative stress condition leading to an increased expression of genes encoding antioxidant enzymes and their activities. Intracellular ANT is the substrate of metabolizing enzymes such as monooxygenases, dioxygenases, polyphenol oxidases, glutathione-*S*-transferases, and sulfotransferases.

The green macroalga *U. lactuca* rapidly incorporated and metabolized ANT reaching a complete degradation after 48 h of culture. ANT induced an oxidative stress condition characterized by the accumulation of ROS, mainly superoxide anions, and lipoperoxides. ANT induced the activation of antioxidant enzymes and the metabolizing enzyme GST and their activation was due, at least in part, by the increase in their expression. Using inhibitors, it was shown that monooxygenases, dioxygenases, GSTs, PPOs and STs participate in ANT metabolization in *U. lactuca* (see model in Fig. 8).

## Materials and methods

### Alga sampling

*Ulva lactuca* was collected from Cachagua (32° 34′ S, 71° 27′ W) a non-polluted site from central Chile, transported in a cooler at 4 °C, manually cleaned from other algae species, sonicated twice using 53 Hz for 2 min to remove epiphytic bacteria, and maintained in artificial seawater (Sigma-Aldrich, St Louis, CA, USA).

### In vitro cultures

*Ulva lactuca* (10 g of fresh tissue) was cultivated in 300 mL of artificial sea water without (control) or with 1, 10, 50, 100 or 250 µM of anthracene (ANT) having a purity ≥ 99% (Sigma-Aldrich, St Louis, CA, USA) for 7 days, at 14 °C with air bubbling and a photoperiod of 14 h light and 10 h darkness, as described in González et al.^24^. Since ANT has low solubility in water, a 25 mM of ANT stock solution was prepared in DMSO with a purity ≥ 99% (Sigma-Aldrich, St Louis, CA, USA) and then an aliquot was dissolved in the culture medium adjusting the DMSO to a final concentration of 0.5% v/v in the medium, including the control cultures. These cultures were used to analyze the accumulation of ANT, for qPCR analyzes, the detection of enzyme activities, and only few fronds were used for cell viability analyzes. All these analyses were performed as independent triplicates.

For assays performed with Phase I and Phase II inhibitors, 10 g of alga were cultivated in 300 mL of artificial seawater without or with the addition of 0.5 µM of mebendazole (MBZ), an inhibitor of CYP450-dependent monooxygenases; cyclohexanedione (CHD), an inhibitor of dioxygenases; 2-naphtoic acid (2-NA), an inhibitor of polyphenol oxidases; ellagic acid (ELA), an inhibitor of GST; and quercetin (QC), an inhibitor of sulfotransferases, and with 5 µM of ANT for 24 h, in triplicates.

### Analysis of cell viability

Three algal laminae were cultivated without ANT and with increasing concentrations of ANT for 7 days and cell integrity and morphology were visualized using Axiovert 100 confocal microscope (Zeiss, Oberkochen, Germany) using an emission wavelength of 488 nm from an argon laser and a filter of 505–550 nm for detection of chlorophyll fluorescence. The images were analyzed using the software LSM510 (Zeiss, Oberkochen, Germany).

### Extraction of anthracene from algae and seawater

The extraction of ANT from algal tissue was performed as described by Sadowska-Rociek et al.^30^, with modifications. One gram of algal dried tissue (DT), which corresponds to 7 g of algal fresh tissue (FT), were pulverized in a mortar with liquid nitrogen and homogenized in 15 mL of cyclohexane having HPLC grade purity (Merck, Darmstadt, Germany). Cellular debris were removed using Quechers AOAC method kit (Agilent Technologies, Santa Clara, CA, USA) and centrifugation at 10,000×*g* for 10 min^31^. Pigments were removed using Quechers SPE dispersive kit (Agilent Technologies, Santa Clara, CA, USA) (Gratz et al.^31^). The extracts recovered (7 mL) were filtered using PVDF filters with 0.2 µm pore size (Finetech, Taichung, Taiwan) and stored in amber glass vials at 4 °C.

Extraction of ANT from artificial seawater was performed as described by Colombo et al.^32^, with modifications. The culture medium (300 mL) was extracted twice with 200 mL of cyclohexane using an extraction funnel, and the extracts were concentrated to 2 mL using a rotatory evaporator HeiVap (Heidolph Industries, Schwabach, Germany). The extracts were filtered using PVDF filters with 0.2 µm pore size (Finetech, Taichung, Taiwan) and stored in amber glass vials at 4 °C.

Extraction of ANT from cultures with inhibitors was performed according to Warshawski et al.^17^, with modifications. Fresh tissue (10 g) was mixed with 250 mL of ethyl acetate HPLC grade purity (Merck, Darmstadt, Germany) and subjected to sonication for 30 min at room temperature. The solvent was collected and concentrated to 5 mL using a rotatory evaporator HeiVap. For the elimination of chlorophyll and pigments, a reaction mixture of 1 mL was prepared with an aliquot of 100 µL of the extract, 150 mM tert-butyl hydroperoxide and 50 mM sodium hydroxide in acetonitrile. The preparation was agitated in vortex for 60 min and then centrifuged at 10,000×*g* for 10 min. The supernatant was recovered and filtered using PVDF filters with 0.2 µm pore size and stored at 4 °C.

### Quantification of anthracene by HPLC

ANT in extracts was analyzed according to Gratz et al.^31^ using an Infinity 1260 series HPLC (Agilent Technologies, Santa Clara, CA, USA) having a reverse phase column Zorbax Eclipse XDB-C18 (4.5 mm × 15 mm and particle size of 5 µm, Agilent Technologies, Santa Clara, CA, USA) and a fluorescence detector. The elution program consisted in a mobile phase of H_2_O/CH_3_CN with a flux of 0.8 mL min^−1^ at 25 °C and a step from 0 to 1.5 min of 40% H_2_O and 60% CH_3_CN, a step from 1.5 to 7 min of 10% H_2_O and 90% CH_3_CN, and a step from 7 to 13 min of 0% H_2_O and 100% CH_3_CN. ANT was detected using excitation wavelength of 260 nm and emission wavelength of 520 nm a at retention time of 6.03 min, representative chromatograms for the standard of ANT and for ANT in tissue and in the culture medium are presented in Supplementary Figure S1.

### Quantification of hydrogen peroxide, superoxide anions and lipoperoxides

Hydrogen peroxide was determined as described in González et al.^33^. Algae (1.5 g of FT) were collected from cultures every 30 min and immediately incubated in 100 mM phosphate buffer pH 7.0 containing 10 µM of 2′,7′-dichlorodihydrofluorescein-diacetate (DCF-DA, Invitrogen, Carlsbad, CA, USA) for 30 min. Algae were rinsed with fresh artificial seawater and dried with tissue paper. The samples were pulverized in a mortar with liquid nitrogen, homogenized in 5 mL Tris–HCl pH 7.0 and centrifuged at 10,000×*g* for 15 min. DCF fluorescence was determined using an excitation wavelength of 480 nm and an emission wavelength of 590 nm using a spectrofluorometer PerkinElmer model LS-5 (PerkinElmer, Waltham, MA, USA) The concentration of hydrogen peroxide was calculated using a calibration curve prepared with 0–1 µM of DCF.

Superoxide ions were determined as described in González et al.^33^. Algae (1.5 g of FT) were collected from cultures every 2 h and immediately incubated in 100 mM phosphate buffer pH 7.0 containing 100 µM hydroethidine (Invitrogen, Carlsbad, CA, USA) for 30 min. Algae were pulverized in a mortar with liquid nitrogen, homogenized in 5 mL Tris–HCl pH 7,0 and centrifuged at 10,000×*g* for 15 min. 2-Hydroxy ethidium (2-HE) fluorescence was detected using an excitation wavelength of 488 nm and an emission wavelength of 525 nm using a spectrofluorometer PerkinElmer model LS-5. The concentration of superoxide ions was calculated using the extinction coefficient of 2-HE (ε = 9.4 mM^−1^ cm^−1^).

Lipoperoxides were determined as described in Ratkevicius et al.^34^. Algae (1 g of FT) were pulverized in a mortar with liquid nitrogen and homogenized with 5 mL of 0.1% v/v of trichloroacetic acid (TCA). The mixture was centrifuged at 10,000×*g* for 20 min, a sample of 200 µL was collected. The sample was mixed with 800 µL of an aqueous solution of 0.5% v/v thiobarbituric acid in 20% m/v TCA and the mixture was incubated at 90 °C for 30 min. The absorbance of malondialdehyde (MDA) adduct was measured at 510 nm using a spectrophotometer model Cary 8520 (Agilent Technologies, Santa Clara, CA, USA). The concentration of lipoperoxides was calculated using the extinction coefficient of MDA adduct (ε = 155 mM^−1^ cm^−1^).

### Preparation of protein extracts

Protein extracts were prepared González et al.^33^. Fresh tissue (6 g) was pulverized in a mortar with liquid nitrogen and homogenized with 30 mL of 100 mM sodium phosphate buffer pH 7.4 containing 5 mM 2-mercaptoethanol and the mixture was centrifuged at 10,000×*g* for 30 min at 4°. The proteins in the supernatant were precipitated with 0.6 g mL^−1^ ammonium sulphate, shaking at 4° for 1 h and then centrifuged at 10,000×*g* for 30 min. Protein pellet was solubilized in 1 mL 100 mM sodium phosphate buffer pH 7.4 containing 20% glycerol and 2 mM 2-mercaptoethanol. Protein concentration was determined according to Bradford^35^.

### Detection of antioxidant enzyme activities

Antioxidant enzyme activities were detected as described in González et al.^24^. Superoxide dismutase (SOD) activity was determined in 1 mL of reaction mixture containing 30 mM Tris–HCl pH 7.0, 0.1 mM EDTA, 20 mM riboflavin, 0.6 mM NBT and without protein extract or with 50 µg of protein extract. The reaction mixture was incubated under white light for 15 min and absorbance of formazan, the reduced form of NBT, was determined at 560 nm. The specific activity of SOD was calculated considering that 1 U of enzyme is the amount of the enzyme that reduces 50% of total NBT which is equal to the amount of dismutated superoxide anions.

Catalase (CAT) activity and other antioxidant enzyme activities were determined according to Ratkevicius et al.^34^ in 1 mL of reaction mix containing 100 mM sodium phosphate buffer pH 7.0, 1 mM hydrogen peroxide and without protein extract or with 30 µg of protein extract. The decrease in absorbance due to consumption of hydrogen peroxide was detected at 240 nm for 2 min. The specific activity of CAT was calculated using a calibration curve prepared with hydrogen peroxide from 1 nM to 1 mM.

Ascorbate peroxidase (AP) activity was determined in 1 mL of reaction mixture containing 100 mM sodium phosphate buffer pH 7.0, 0.4 mM ascorbate, 1 mM hydrogen peroxide and without protein extract or with 20 µg of protein extract. The decrease in absorbance due to the consumption of ascorbate was detected at 290 nm for 1 min. The specific activity was calculated using the molar extinction coefficient of ascorbate (ε = 2.8 mM^−1^ cm^−1^).

Dehydroascorbate reductase (DHAR) activity was determined in 1 mL of reaction mixture containing 100 mM sodium phosphate buffer pH 7.0, 1 mM glutathione, 0.5 mM of dehydroascorbate, and 50 µg of protein extract. The increase in absorbance due to production of ascorbate was detected at 290 was for 2 min. The specific activity was calculated using the molar extinction coefficient of ascorbate (ε = 2.8 mM^−1^ cm^−1^).

Glutathione reductase (GR) activity was determined in 1 mL of reaction mix containing 100 mM sodium phosphate buffer pH 7.0, 0.5 mM GSSG, 0.15 mM NADPH and 20 µg of protein extract. The decrease in absorbance due to consumption of NADPH was detected at 340 nm for 1 min. The specific activity was calculated using the NADPH molar extinction coefficient of NADPH (ε = 6.2 mM^−1^ cm^−1^).

Glutathione peroxidase (GP) was determined in 1 mL of reaction mix containing 100 mM sodium phosphate buffer pH 7.0, 0.5 mM glutathione, 0.15 mM NADPH, 1 U glutathione reductase, 1 mM hydrogen peroxide and without protein extract or with 20 µg of protein extract. The decrease in absorbance due to consumption of NADPH was detected at 340 nm for 2 min. The specific activity was calculated using the NADPH molar extinction coefficient of NADPH (ε = 6.2 mM^−1^ cm^−1^).

### Detection of detoxification enzyme

Detection of detoxification enzymes was performed as described in González et al.^24^. Glutathione-*S*-transferase (GST) activity was determined in 1 mL of reaction mix containing 100 mM sodium phosphate buffer pH 7.0, 0.5 mM GSH, 1 mM CDNB and 50 µg of protein extract. The increase in absorbance due the formation of the adduct GSH-CDNB was detected at 340 nm 2 min. The specific activity was calculated using molar extinction coefficient of the adduct (ε = 9.6 mM^−1^ cm^−1^).

### RNA extraction and qPCR analysis

RNA extraction and qPCR analysis was performed as described in González et al.^24^. Total RNA was extracted from 50 mg of fresh tissue using the FavorPrep Plant Total RNA mini kit (Favorgen, Ping-Tung, Taiwan). Total RNA (2 µg) was used to synthesize cDNA using the Affinityscript qPCR cDNA synthesis kit (Agilent Technologies, Santa Clara, CA, USA). Real time PCR was performed with 50 ng of cDNA, 400 nM of each primer and using Brilliant III ultra-fast SYBR green qPCR kit (Agilent Technologies, Santa Clara, CA, USA) and an Aria MX real-time PCR system (Agilent Technologies, Santa Clara, CA, USA). The amplification program consisted of an initial step of 3 min at 95 °C and 40 cycles of 5 s at 95 °C followed by 10 s at 55 °C. Primers to detect SOD, AP, CAT, GR, GST transcripts were designed using sequences obtained from *U. intestinalis*, *U. fasciata* and *U. compressa* (NCBI GeneBank, *U. compressa* SRA records SRP145672 and PRJNA557176). Primers were: superoxide dismutase (GeneBank EF437244.1) SOD-F 5′CCT GCT CAG GCA ACC TCC TT3′, SOD-R 5′TGC CAG CCT TCC AGT CTC AC3′, catalase (GeneBank DQ286544.1) CAT-F 5′ACC AAG AGG CGG AGA AAG TG3′, CAT-R 5′CGA AGT CAA ACC GGT CCT CA3′, ascorbate peroxidase (GeneBank DQ286543.1) AP-F 5′CCA CAA GGC TGA GAC CAA GT3′, AP-R 5′CTT TGT CCA CTC AGG GGT CC3′, dehydroascorbate reductase (GeneBank XM_001698323.1) DHAR-F 5′GCG ACT CGT ACT GCT CCT AC3′, DHAR-R 5′GAA AAC GGA ACT CCG CGC TT3′, glutathione reductase (GeneBank DQ286546.1) GR-F 5′AGT TCG GGG ACG TGG ATG TG3′, GR-R 5′CTC TCG TTG CCG GAG ATC GT3′, glutathione peroxidase (GeneBank XM_001698523.1) GPX-F 5′CAC GCG TCC GGT AAC ACT3′, GPX-R 5′GGG ACG AGA AAG GTT GCT GA3′. Glutathione *S*-transferase (GeneBank FD387475.1) GST-F 5′GGA CCC ATG CAA CGC CAA G3′, GST-R 5′GGT GCG CTG TAC ACC CAA GA3′, and α-tubulin (GeneBank EU701065.1) as housekeeping transcript, TUB-F 5′ACA GGC TCA TTG CTC AGG TC3′, TUB-R 5′TCT CGG CAG AGA TGA CTG GA3’.

### Statistical analysis

Experimental results were subject to one-way analysis of variance (ANOVA) and post hoc Tukey Multiple Comparison Test using the statistical software Prism 6 (Graph Pad software Inc., San Diego, CA, USA), previous to the evaluation of the requirements of normality and homogeneity of variance. Significant differences were estimated using three independent replicates at a 95% confidence interval.

## Supplementary Information

Supplementary Information 1.
