# Supplementary figures and images for: Anthracene induces oxidative stress and activation of antioxidant and detoxification enzymes in Ulva lactuca (Chlorophyta)
Source: Sci Rep. 2021 Apr 8;11:7748. doi: 10.1038/s41598-021-87147-5 (PMC8032757; doi:10.1038/s41598-021-87147-5)

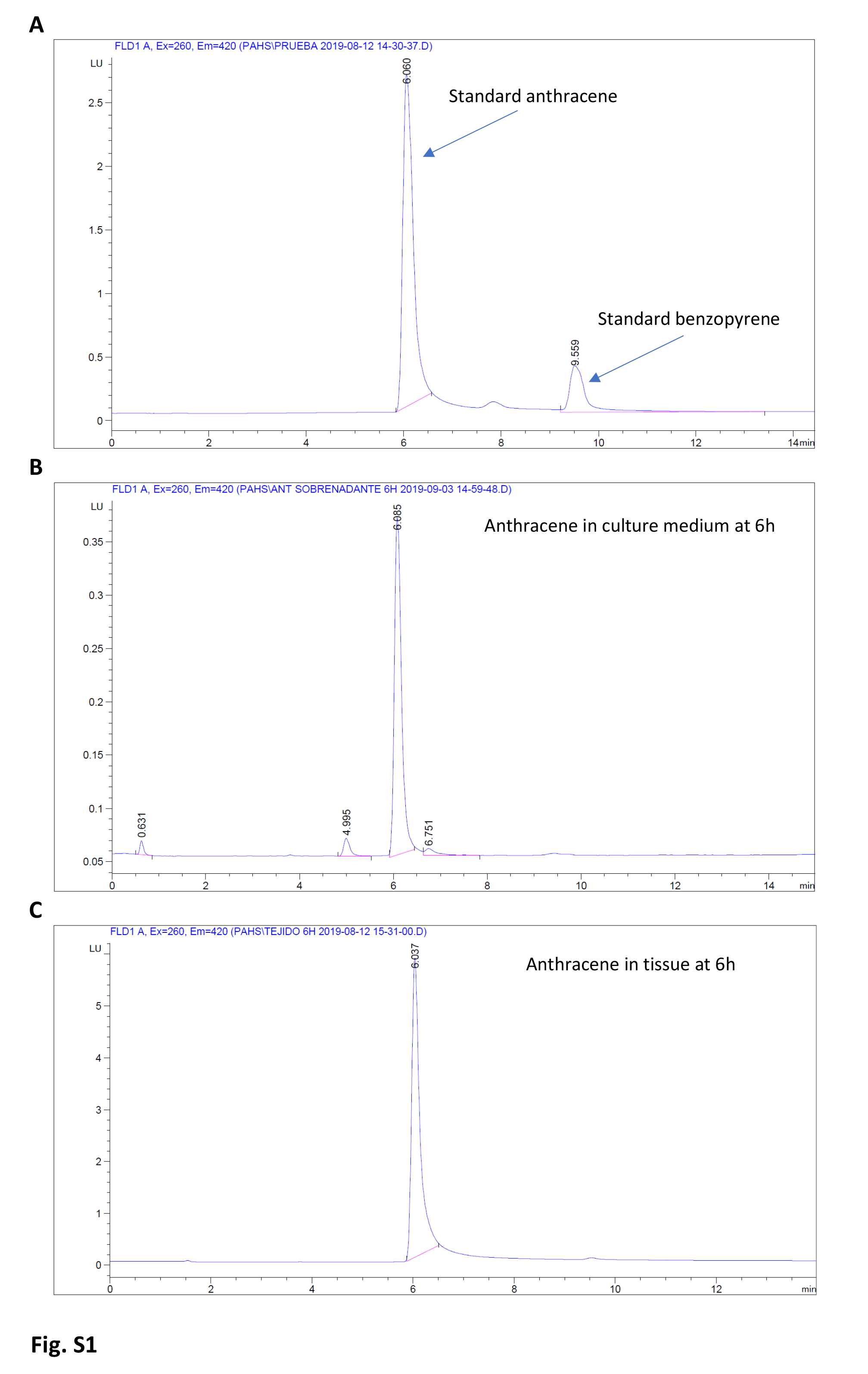

Supplement: Supplementary file 1 — Supplementary Information 1. [file 41598_2021_87147_MOESM1_ESM.tif]
